# Treatment of unresectable, locally advanced pancreatic adenocarcinoma with combined radiochemotherapy with 5-fluorouracil, leucovorin and cisplatin

**DOI:** 10.1054/bjoc.1999.0884

**Published:** 1999-12-08

**Authors:** G V Kornek, A Schratter-Sehn, A Marczell, D Depisch, J Karner, G Krauss, K Haider, W Kwasny, G Locker, W Scheithauer

**Affiliations:** Division of Oncology, Department of Internal Medicine I, Vienna University Medical School, Waehringer Guertel 18-20, Vienna, A-1090, Austria; Departments of Radiation Oncology, General Surgery, Kaiser-Franz-Josef Hospital, Kundratstrasse 3, Vienna, A-1100, Austria; Department of Surgery, Hanusch Hospital, Heinrich-Collin-Strasse 30, Vienna, A-1140, Austria; Department of Surgery, Wr. Neustadt General Hospital, Corvinusring 3-5, Wr. Neustadt, A-2700, Austria; Department of Surgery, Stockerau General Hospital, Landstrasse 16-18, Stockerau, A-2000, Austria

**Keywords:** pancreatic cancer, neoadjuvant, chemoradiation, cisplatin, 5-fluorouracil, leucovorin

## Abstract

The aim of the study was to evaluate the effectiveness and safety of a combined treatment modality including systemic chemotherapy with 5-fluorouracil (FU), leucovorin, cisplatin and external beam radiotherapy in patients with locally advanced pancreatic cancer. Systemic chemotherapy consisted of FU 400 mg m^−2^and leucovorin 20 mgm^−2^both given as intravenous bolus injection on days 1–4, plus cisplatin 20 mgm^−2^administered as 90-min infusion on days 1–4. Treatment courses were repeated every 4 weeks × 6 unless prior evidence of progressive disease. Radiation therapy using megavolt irradiation of ≥ 6 MV photons with a 3- or 4-field technique was delivered during the second and third chemotherapy course, that was reduced in dose by 25%. Between October 1994 and July 1996, a total of 38 patients were entered onto this trial, all of whom were assessable for toxicity and survival. Eighteen of these (47%) had objective remissions to combined radiochemotherapy, including four CR (11%), 13 (34%) had stable disease and seven patients (18%) showed tumour progression during treatment. The median progression-free interval of the entire study population was 10 months (range 3–32), and median overall survival was 14.0 months (range 3–45+ months); 53% of all patients were alive at 12 months, and 18% of patients were alive at 24 months respectively. Severe haematological side-effects comprised neutropenia in 18%, thrombocytopenia in 8% and anaemia in 11%. The most frequent non-haematological side-effects were nausea/vomiting (WHO grade 3: 18%), and diarrhoea (grade 3: 13%). This combined radiochemotherapy regimen was tolerable and effective in patients with locally advanced pancreatic cancer. Since therapeutic results, in fact, compare favourably with other series, including surgical treatment of potentially resectable tumours, further evaluation of combined treatment modalities in the neoadjuvant setting seems warranted. © 2000 Cancer Research Campaign


*British Journal of Cancer* (2000) **82**(1), 98–103
© 2000 Cancer Research Campaign
Article no. bjoc.1999.0884

### Treatment of unresectable, locally advanced pancreatic adenocarcinoma with combined radiochemotherapy with 5-fluorouracil, leucovorin and cisplatin

**GV Kornek[1], A Schratter-Sehn[2], A Marczell[4], D Depisch[5], J Karner[3], G Krauss[6], K Haider[5], W Kwasny[5], G Locker[1] and W Scheithauer[1]**

[1]Division of Oncology, Department of Internal Medicine I, Vienna University Medical School, Waehringer Guertel 18-20, A-1090 Vienna, Austria; [2]Departments of Radiation Oncology and [3]General Surgery, Kaiser-Franz-Josef Hospital, Kundratstrasse 3, A-1100 Vienna, Austria; [4]Department of Surgery, Hanusch Hospital, Heinrich-Collin-Strasse 30, A-1140 Vienna, Austria; [5]Department of Surgery, Wr. Neustadt General Hospital, Corvinusring 3-5, A-2700 Wr. Neustadt, Austria; [6]Department of Surgery, Stockerau General Hospital, Landstrasse 16-18, A-2000 Stockerau, Austria

**Summary** The aim of the study was to evaluate the effectiveness and safety of a combined treatment modality including systemic chemotherapy with 5-fluorouracil (FU), leucovorin, cisplatin and external beam radiotherapy in patients with locally advanced pancreatic cancer. Systemic chemotherapy consisted of FU 400 mg $m^{-2}$ and leucovorin 20 mg $m^{-2}$ both given as intravenous bolus injection on days 1–4, plus cisplatin 20 mg $m^{-2}$ administered as 90-min infusion on days 1–4. Treatment courses were repeated every 4 weeks $\times$ 6 unless prior evidence of progressive disease. Radiation therapy using megavolt irradiation of $\geq$ 6 MV photons with a 3- or 4-field technique was delivered during the second and third chemotherapy course, that was reduced in dose by 25%. Between October 1994 and July 1996, a total of 38 patients were entered onto this trial, all of whom were assessable for toxicity and survival. Eighteen of these (47%) had objective remissions to combined radiochemotherapy, including four CR (11%), 13 (34%) had stable disease and seven patients (18%) showed tumour progression during treatment. The median progression-free interval of the entire study population was 10 months (range 3–32), and median overall survival was 14.0 months (range 3–45+ months); 53% of all patients were alive at 12 months, and 18% of patients were alive at 24 months respectively. Severe haematological side-effects comprised neutropenia in 18%, thrombocytopenia in 8% and anaemia in 11%. The most frequent non-haematological side-effects were nausea/vomiting (WHO grade 3: 18%), and diarrhoea (grade 3: 13%). This combined radiochemotherapy regimen was tolerable and effective in patients with locally advanced pancreatic cancer. Since therapeutic results, in fact, compare favourably with other series, including surgical treatment of potentially resectable tumours, further evaluation of combined treatment modalities in the neoadjuvant setting seems warranted. © 2000 Cancer Research Campaign

**Keywords** pancreatic cancer; neoadjuvant; chemoradiation; cisplatin; 5-fluorouracil, leucovorin

The incidence of carcinoma of the exocrine pancreas has increased over the last decades, and it is now the fourth leading cause of cancer death worldwide (Parker et al, 1996). Despite certain advances in diagnosis, surgical procedures and chemotherapy, the prognosis of pancreatic cancer remains poor. Resection of the tumour at an early stage of the disease is the only curative treatment option. Unfortunately, as pancreatic cancer lacks early symptoms, less than 25% of patients undergo complete resection. The 5-year survival of these patients has been reported to be less than 5%, and most of them experience local failure that often produces debilitating complications, such as pain, jaundice, duodenal obstruction, malnutrition and haemorrhage (Tepper et al, 1976; Griffin et al, 1990; Warshaw et al, 1992; Gudjonsson et al, 1995).

Therefore, more effective treatment modalities are needed to increase the number of resectable tumours and to reduce local failure. In 1987, the Gastrointestinal Tumor Study Group (GITSG) demonstrated that post-operative chemotherapy combined with external-beam radiation results in superior survival of patients with pancreatic cancer after curative resection compared to surgery alone (Gastrointestinal Tumor Study Group, 1987). The magnitude of the operation and its associated morbidity, however, often results in a lengthy period of recovery, which represents a major obstacle to the routine use of post-operative chemoradiation (Yeo et al, 1995; Spitz et al, 1997). Accordingly, several more recent trials have been conducted to investigate the impact of preoperative chemoradiation. The rationale for the addition of chemotherapy to irradiation is that cytostatic drugs are able to enhance the effect of radiation therapy. Combined treatment modality, when given preoperatively, can also shrink tumour size and a greater proportion of patients with locally advanced disease may undergo curative resection.

Despite the availability of more than 50 active chemotherapeutic agents, only few single agents or combinations of cytotoxic drugs have demonstrated activity against this tumour (Schnall et al, 1996).

5-Fluorouracil (5-FU) is the most commonly used single agent with a median response rate of less than 20%. Inhibition of thymidilate synthase, the target enzyme of FU, can be enhanced by leucovorin (LV) because this drug increases the cytosolic levels of reduced folates (Spitz et al, 1997). Various in vitro investigations

*Received 25 January 1999*
*Revised 26 June 1999*
*Accepted 8 July 1999*
*Correspondence to:* GV Kornek and clinical trials showed that cisplatin (CDDP) consists of a non-cross-resistant, non-overlapping toxic activity, and exerts a different and/or further synergistic mechanism of action as fluorouracil. 5-FU, LV plus cisplatin is a drug combination with established anticancer activity in head-and-neck, oesophageal and anal carcinoma (Schnall et al, 1996). The limited therapeutic value of available chemotherapeutic drug combinations in pancreatic malignancies and the documented synergistic activity of 5-FU/LV/CDDP, which might be further enhanced by simultaneous radiation therapy, prompted us to initiate the present phase II trial. The goal of our study was to evaluate response rate, overall survival and tolerance of chemoradiation with this regimen in previously untreated patients suffering from locally advanced pancreatic cancer.

### PATIENTS AND METHODS

### Eligibility criteria

Patients with histologically or cytologically confirmed unresectable stage III and IVA adenocarcinoma of the pancreas were enroled onto this trial between January 1994 and October 1996. They were required to have a World Health Organization (WHO) performance status of 0–2, age between 18 and 75 years, life expectancy of at least 3 months, and adequate bone marrow (WBC count $\geq$ 4000 $\mu l^{-1}$, platelet count $\geq$ 100 000 $\mu l^{-1}$), renal (serum creatinine concentration < 1.5 mg $dl^{-1}$) and hepatic functions (serum bilirubin < 1.5 mg $dl^{-1}$, serum transaminase level < 2 $\times$ of the upper normal range). Patients were staged by laparotomy and open biopsy, or by fine-needle aspiration cytology following computerized tomographic (CT) scanning and angiography as needed to document unresectable disease. Endoscopic ultrasonography was also used in the more recent cases. Staging followed the American Joint Committee (AJCC) tumour-node-metastasis system (Fleming et al, 1997). All patients had to have measurable disease that could be assessed by radiographic procedures. Patients were excluded if they had distant metastases, serious or uncontrolled concurrent medical illness, known peripheral polyneuropathy or a history of other malignancies. No prior chemotherapy or radiation therapy was allowed. Informed consent was obtained from all patients according to institutional regulations, and the study was approved by the local ethics committee.

Pretreatment evaluation included a complete medical history, physical examination, complete blood cell count, differential blood cell count, biochemistry analysis, chest X-ray, electrocardiogram (ECG) and CT of the abdomen.

### Treatment protocol

#### *Chemotherapy*

Systemic chemotherapy consisted of LV 20 mg $m^{-2}$, 5-FU 400 mg $m^{-2}$, both given as intravenous bolus injection, and cisplatin 20 mg $m^{-2}$ given as 90-min infusion. All drugs were administered on 4 consecutive days, and treatment courses were repeated every 4 weeks. Sufficient hydration to ensure a urinary output of at least 100 ml $h^{-1}$ before and 4 h after the infusion of cisplatin was required. The administration of mannitol or loop diuretics was left to the discretion of the investigator. Concomitant medications routinely given before cytotoxic drug administration included 8 mg ondansetron and 8 mg dexamethasone. The dose of all chemotherapeutic drugs was reduced by 25% during concurrent radiotherapy (second and third course), and if a patient experienced WHO grade 3 organ and/or grade 4 haematological toxicity during the previous cycle. Elevations of the serum creatinine level to $\geq$ 150% of the pretreatment value resulted in discontinuation of cisplatin for one course of the therapy, with resumption only after return of the serum creatinine to its baseline value. If nephrotoxicity persisted at the start of the next cycle of therapy, cisplatin was withheld. Patients continued to receive their assigned treatment for a total of six courses, provided that they did not develop progressive disease.

#### *Radiation therapy*

During the second and third chemotherapy cycle, radiotherapy was delivered using megavolt irradiation of $\geq$ 6 MV photons, most commonly 10 MV photons, with a three- or four-field technique. The daily fraction of radiation was 1.8 Gy given 5 days a week to a prescribed total dose of 55 Gy. Treatment volumes encompassed the primary tumour, as defined on CT-scans or by clips placed at the time of surgery, and the areas of potential nodal involvement with at least a 3 cm margin in all directions covering the pancreaticoduodenal, porta hepatic and celiac axis lymph nodes for the initial 45 Gy, followed by a conedown field to the gross tumour with a margin of 2 cm. Computer-assisted simulation programmes were used routinely. Treatment was individualized based on the volume and location of disease. To counteract or avoid nephrotoxicity and haematological complications, the radiation field was required to spare the left kidney, and half of the right kidney and the spinal cord dose was limited to 4000 Gy or less.

Surgical resection of the tumour was not part of the original protocol; however, it was to be considered 4–6 weeks after completation of combined radiochemotherapy in responding patients, provided that they did not initially present with involvement of major vessels and that they met all clinical and radiological criteria for resectability as assessed by a CT and angiography reevaluation.

### Toxicity and response criteria

Toxicity was evaluated according to WHO standard criteria. Haematological parameters were assessed every 2 weeks, and all other adverse reactions were evaluated retrospectively before the next cycle. For response evaluation, CT reassessments were repeated every 8 weeks Objective response had to be confirmed in one subsequent examination after a 4-week interval. A complete response (CR) was defined as a total resolution of all evidence of tumour without appearance of new lesions on two consecutive evaluations 4 weeks apart. A partial response (PR) required a 50% reduction in the maximum perpendicular tumour measurements, with no new lesion appearing for at least 4 weeks. No change (NC) was defined as less than 50% reduction and less than 25% increase of measurable tumour lesions lasting for at least 8 weeks. Patients were considered to have progressive disease (PD) if the measurable tumour lesions increased by greater than 25% according to initial staging or if new lesions appeared within the first 2 months of therapy. For patients who underwent resection after treatment, the pathologic residual tumour was correlated with the pretreatment tumour mass. Survival was determined from the date of first treatment until death or until the patient was last examined alive. Time to progression was determined as the interval between the date of first treatment and the date PD was first observed.

© 2000 Cancer Research Campaign

**Table 1** Patient characteristics

| | |
|---|---|
| No. of patients | 38 |
| Age in years | |
| Median | 60 |
| Range | 30–70 |
| Sex | |
| Female | 22 |
| Male | 16 |
| WHO performance status | |
| 0 | 18 |
| 1 | 16 |
| 2 | 4 |
| Clinical stage | |
| III | 26 |
| IVA | 12 |
| Histological grade | |
| G1 | 7 |
| G2 | 16 |
| G3/4 | 9 |
| GX | 6 |
| Location of primary tumour | |
| Head | 25 |
| Head and body | 7 |
| Body | 4 |
| Tail | 2 |
| Prior surgery | |
| None | 6 |
| Explorative | 8 |
| Palliative bypass | 24 |

WHO, World Health Organization.

**Table 2** Maximum toxicity in 38 patients treated with combined RCT

| Type of toxicity (WHO grade) | Number of patients at toxicity (%) 1 | 2 | 3 | 4 |
|---|---|---|---|---|
| *Haematological* | | | | |
| Leukopenia | 17 (45%) | 16 (42%) | 6 (16%) | – |
| Granulocytopenia | 11 (29%) | 15 (39%) | 4 (11%) | 3 (8%) |
| Thrombocytopenia | 9 (24%) | 11 (29%) | 3 (8%) | – |
| Anaemia | 16 (42%) | 5 (13%) | 3 (8%) | 1 (3%) |
| *Non-haematological* | | | | |
| Nausea/vomiting | 7 (18%) | 5 (13%) | 7 (18%) | – |
| Stomatitis | 4 (11%) | 4 (11%) | – | – |
| Diarrhoea | 3 (8%) | 6 (16%) | 5 (13%) | – |
| Constipation | 3 (8%) | – | – | – |
| Abdominal pain | 3 (8%) | 3 (8%) | – | – |
| Anorexia | 5 (13%) | 4 (11%) | – | – |
| Alopecia | 3 (8%) | 1 (3%) | 3 (8%) | – |
| Fatigue | 4 (11%) | 6 (16%) | – | – |
| Peripheral neuropathy | 3 (8%) | – | – | – |
| Infection | 5 (13%) | 3 (8%) | – | – |
| Liver | 3 (8%) | – | – | – |
| Kidney | 5 (13%) | – | – | – |

**Table 3** Response to treatment

| Variable | No. of patients (%) |
|---|---|
| Complete response | 4 (11%) |
| Partial response | 14 (37%) |
| Stable disease | 13 (34%) |
| Progression | 7 (18%) |
| Overall response rate | 18/38 (47%) |
| 95% confidence interval | 31–64% |
| Time to progression, months | |
| Median | 10.0 |
| Range | 3–35 |
| Survival, months | |
| Median | 14.0 |
| Range | 3–45+ |
| 1-year-survival | 20 (53%) |
| 2-year-survival | 7 (18%) |

### Statistical analysis

Survival and progression-free survival were calculated by the Kaplan–Meier product method (Kaplan and Meier, 1958). Survival curves for prognostic factors were compared by the log-rank test for censored observations (BMDP Statistical Software, 1985). Confidence intervals were calculated at the 95% confidence level. All patients' records were examined by independent reviewers, and all patients who entered the study were analysed.

### RESULTS

### Patient characteristics

Between October 1994 and July 1996 a total of 38 patients with locally advanced pancreatic cancer were entered onto this trial. Their pretreatment characteristics are listed in Table 1. Twenty-two patients were female and 16 were male, their median age was 60 years, and the median WHO performance status was 1. Twenty-five patients had carcinomas located in the head of the pancreas, seven had them in the head and extending into the body of the pancreas, four patients had primary body tumours, and two tumours were located in the tail of the pancreas. The size of the primary tumours ranged from 2 cm to 9 cm with a median of 4 cm. Previous surgery included palliative digestive and/or biliary anastomosis in 24 patients, and explorative laparatomy in eight. Only six patients had a fine-needle aspiration biopsy for diagnosis without laparotomy.

### Treatment summary

A total of 181 chemotherapy courses were administered to the 38 patients with a median of five courses per patient (range 2–6). Cytotoxic drug doses were lowered according to the study protocol to counteract grade 4 haematotoxicity in four patients (11%), and severe gastrointestinal side-effects (nausea and vomiting and/or diarrhoea) in six patients. Twelve patients (32%) had at least one treatment delay of 1 week some time during therapy, and the total number of delayed courses was 15. The reasons for delayed courses were haematological in seven, protracted nausea/vomiting in three and/or diarrhoea in five cases. In three patients, who suffered from protracted vomiting and diarrhoea, however, the underlying reason was found to be tumour progression with infiltration of the duodenum and peritoneal carcinomatosis. Radiotherapy was initiated in all 38 patients, and 34 (89%) completed the planned treatment course. The median dose of radiation therapy was 51 Gy (range 12–56 Gy). Four patients did not complete external-beam radiation; three of them had rapid tumour progression and one patient was discontinued because of protracted thrombo- and granulocytopenia. Radiation suspension occurred in seven patients due to diarrhoea ($n$ = 4) and/or vomiting ($n$ = 1) and/or haematological toxicity ($n$ = 2).

© 2000 Cancer Research Campaign

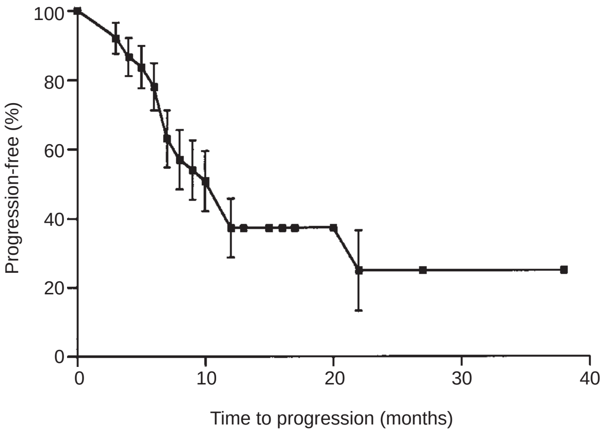

**Figure 1** Time to progression curve for the entire patient group (*n* = 38). The median time to progression was 10.0 months

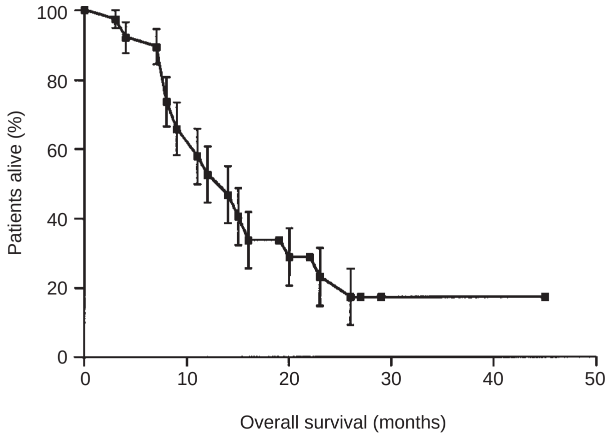

**Figure 2** Survival curve for the entire patient group (*n* = 38). The median survival time was 14.0 months, with a 53% 1-year survival rate

### Toxicity

Table 2 summarizes the entire experience of worst-ever toxicities during all treatment courses with and without concomitant radiation therapy. Haematological toxicity was frequent, but generally mild to moderate. Only four patients (11%) experienced grade 3 and three patients (7%) grade 4 neutropenia, and there were no hospitalizations for granulocytopenic fever; the lowest median absolute granulocyte count was 2630 $\mu l^{-1}$ (range 400–16 600 $\mu l^{-1}$). Grade 3 thrombocytopenia occurred in three patients (7%), and the median platelet count nadir was 148 000 $\mu l^{-1}$ (range 42 000–619 000 $\mu l^{-1}$). Four patients required red blood cell transfusion due to symptomatic anaemia, which was grade 3 in three and grade 4 in one respectively. The most frequently encountered non-haematological adverse reactions were nausea and vomiting (49%), which were rated mild to moderate in 12 patients, and severe in seven. Other common gastrointestinal side-effects included diarrhoea in 34% (grade 3: five patients), and mild to moderate mucositis in eight patients (20%). Only three patients experienced CDDP-related mild and fully reversible peripheral neuropathy, and temporary renal toxicity occurred in five patients (13%). Overall, adverse reactions due to irradiation were tolerable and fully reversible, and included vomiting and diarrhoea, as well as abdominal pain in six patients.

### Therapeutic results and patient outcome

The overall response rate was 47% (95% CI 31–64%), including four complete (11%) and 14 partial remissions. In 13 patients (34%) the tumour was rated stable, and in seven patients (18%) disease progressed. Only 3/18 patients with objective response underwent surgical exploration (17%). In all of them potentially curative pancreaticoduodenectomy could be accomplished. One patient had a histopathologically confirmed complete remission, and in the two other patients complete resection of the residual tumour was confirmed at staging of the resected specimens. Of the responders who did not undergo surgery, the reasons were documented inoperability due to infiltration/encroachment of the adjacent large vessels prior to initiation of radiochemotherapy in eight patients, refusal for surgical exploration in five, or not being offered surgery because of comorbid medical conditions in two (pre-existing cardiopulmonary disease and recurrent thrombophlebitis in one patient each).

The median progression-free interval was 10 months (range 3–38 months), and the median survival duration was 14.0 months (range 3–45+ months). The overall 1- and 2-year survival rates were 53% and 18% respectively. The survival of the three patients who underwent surgical resection following combined radiochemotherapy was 4, 11 and 12 months. One of these patients died due to pulmonary embolism, and the two others expired due to distant metastatic disease recurrence. There was no recognizable impact of prior surgical staging, tumour grade, haematological or biochemical parameters on overall or progression-free survival.

### DISCUSSION

Disappointing results with surgery, chemotherapy and radiotherapy used individually for stage II and III pancreatic cancer have stimulated clinical trials of combined modality therapy in these patients. Many of these studies demonstrated that both radiation and chemotherapy are necessary to achieve best survival (Nagai et al, 1986; Willet et al, 1993; Gastrointestinal Tumor Study Group, 1979, 1987, 1988). In view of the reported long-term results of several contemporary trials, however, further improvements are certainly warranted (Forastiere et al, 1990; Kompki et al, 1992; Wagener et al, 1992).

The chemotherapeutic drug combination that we have decided to use in the present trial has been shown to be tolerable and active against various solid tumours, including pancreatic carcinoma as indicated in a recent phase II study in patients with metastatic disease (Hart et al, 1989; Dreyfuss et al, 1990; Vokes et al, 1992; Scheithauer et al, 1994; Andre et al, 1996). 5-FU is the most commonly used cytotoxic drug with an objective response rate of 10–20%. Its anti-tumour activity can be enhanced with addition of radiotherapy as demonstrated in the preclinical and clinical setting (Byfield et al, 1974; Nakyajima et al, 1979; Moertel et al, 1981). The concept of biochemical modulation of 5-FU with LV has not been found successful in patients with metastatic carcinoma of the pancreas (Bruckner et al, 1988; De Caprio et al, 1989; Crown et al, 1991; Weinermann et al, 1994), however, its potential efficacy might have been obscured by the bulk of tumour burden in these patients. Three recently published trials using radiochemotherapy with 5-FU/LV in locally advanced pancreatic tumours, in fact, have demonstrated encouraging results with prolonged survival

© 2000 Cancer Research Campaign

(Moertel et al, 1994; Mohiuddin et al, 1995; Prott et al, 1997). The use of cisplatin in the regimen was based on preclinical evidence that CDDP is a potent radiosensitizer and on its successful addition to 5-FU ± radiation in the treatment of pancreatic and other malignancies, including squamous cell carcinoma of the head and neck, oesophageal and anal carcinoma (Rothman et al, 1991; Nicolson et al, 1995; Hörmann, 1996; Popescu et al, 1997).

The overall response rate of 47% in the present trial, including four radiological complete responses and one pathological complete response, demonstrates that this combination of two potential radiosensitizers with concomitant radiotherapy provides an active regimen for the treatment of patients with locally advanced pancreatic carcinoma. The local control of the primary tumour site was effective and durable: none of the three surgically explored and curatively resected patients, and only one out of the remaining 12 responders progressed within the radiation field. Among patients with stable disease, there were also only two cases, who failed locally. Similarly, the 1- and 2-year survival rates (53% and 18%) seem encouraging and at least comparable with other series, although we were able to achieve these results in a study population including patients who were likely to have been excluded from other trials of preoperative radiochemotherapy because of primarily inoperable disease (Klaasen et al, 1985; Forastiere et al, 1990; Kompki et al, 1992; Wagener et al, 1992; Yeung et al, 1993; Moertel et al, 1994; Mohiuddin et al, 1995; Kamthan et al, 1997; Prott et al, 1997; Hoffman et al, 1998).

The fact that a number of our patients initially presented with portal vein occlusion or infiltration of mesenterial vessels, and/or involved regional lymph nodes, also contributed to the low rate of surgical explorations despite the high objective response rate obtained with this combined modality therapy. Probably, we have also underestimated the potential rate of technically feasible resections, because some additional patients with evidence of residual tumour on imaging studies rated stable may actually have had fibrosis or significantly reduced residual tumour volume and were resectable. Such experience has been reported in neoadjuvant trials of non-small-cell lung and pancreatic cancers with less advanced and resectable disease (Gralla, 1988; Yeung et al, 1993; Evans et al, 1994).

Another important positive feature of this combined radiochemotherapy regimen with 5-FU/LV/CDDP was its tolerance, with 89% of patients completing treatment. Grade 4 myelosuppression was seen in 8%, and severe gastrointestinal side-effects, requiring dose attenuations, occurred in only 13%. There were no life-threatening toxicities, and no treatment-related deaths occurred.

Based on our results in patients with initially unresectable, locally advanced pancreatic adenocarcinoma, this chemoradiotherapy regimen deserves further evaluation as neoadjuvant treatment for earlier stages. Despite sophisticated diagnostic techniques, the majority of patients prove to be unresectable, and exploratory surgery often only delays treatment. As demonstrated in a recent trial involving 142 patients with localized tumours of the pancreatic head, preoperative chemoradiation followed by resection resulted in a similar therapeutic outcome when compared with primary resection followed by adjuvant chemoradiation (Spitu et al, 1997). Twenty-six per cent of the patients, who were found to have disseminated disease after completing preoperative treatment, however, were spared an unnecessary laparotomy. Another advantage of the preoperative approach was related to the fact that all patients received all components of the multimodality regimen in contrast to adjuvant therapy, which in agreement with other series, could not be delivered in one fourth of eligible patient due to prolonged recovery after pancreaticoduodenectomy (Yeo et al, 1995; Spitz et al, 1997).

Further improvements in combined modality treatment might be achieved by using rapid-fractionation radiotherapy implicating the advantage of a much shorter duration of treatment, and by establishing new drugs and combinations that are more effective in counteracting systemic tumour spread which, in line with our experiences, remains responsible for the limited survival duration in patients with pancreatic cancer (Spitz et al, 1997).

### ACKNOWLEDGEMENTS

This study was supported in part by the Austrian Cancer Society/Section of Niederoesterreich and the ‘Gesellschaft zur Erforschung der Biologie und Behandlung von Tumorkrankheiten’

### REFERENCES

Andre T, Lotz JP, Bouleuc C, Azzouzi K, Houry S, Hannoun L, See J, Esteso A, Avenin D and Izrael V (1996) Phase II trial of 5-fluorouracil, leucovorin and cisplatin for treatment of advanced pancreatic adenocarcinoma. *Ann Oncol* **7**: 173–178

BMDP Statistical Software (1985) BMDP Statistical Software Manual. University of California Press: Berkeley, CA

Bruckner HW, Crown J, McKenna A and Hart R (1988) Leucovorin and 5-fluorouracil as a treatment for disseminated cancer of the pancreas and unknown primary tumors. *Cancer Res* **48**: 5570–5572

Byfield JE, Chan PYM and Seagren S (1974) Radiosensitization by 5-fluorouracil (5-FU): molecular and clinical scheduling implications. *Proc Am Assoc Cancer Res* **18**: 74 (abstract)

Crown J, Caspers ES, Botet J, Murray P and Kelsen DP (1991) Lack of efficacy of high-dose leucovorin and fluorouracil in patients with advanced pancreatic. *J Clin Oncol* **9**: 1682–1686

De Caprio JA, Arbuck SG and Mayer RJ (1989) Phase II study of weekly 5-fluorouracil (5-FU) and folinic acid (FA) in previosly untreated patients with unresectable measurable pancreatic adenocarcinoma. *Proc Am Soc Clin Oncol* **8**: 100, (abstract)

Dreyfuss AI, Clark JR, Wright JE, Norris CM, Busse PM, Lucarini JW, Fallon BG, Casey D, Anderson JW and Klein R (1990) Continuous infusion high-dose leucovorin with 5-fluorouracil and cisplatin for untreated stage IV carcinoma of the head and neck. *Ann Intern Med* **112**: 167–172

Evans DB, Abbruzzese J, Lee J, Cleary K and Rich T (1994) Preoperative chemoradiation and pancreaticoduodenectomy for adenocarcinoma of the pancreas. *Proc Am Soc Clin Oncol* **13**: 226 (abstract)

Fleming ID, Cooper JS, Henson DE, Hutter RVP, Kennedy BJ, Murphy GP, O’Sullivan B, Sobin LH and Yarbro JW (eds) (1997) *Exocrine Pancreas*. AJCC Cancer Staging Manual, 5th Edn, pp. 121–126. Lippincott-Raven: Philadelphia

Forastiere AA, Orringer MB, Perez-Tamayo C, Urba SG, Husted S, Takasugi BJ and Zahurak M (1990) Concurrent chemotherapy and radiation therapy followed by transhiatal esophagus. *J Clin Oncol* **8**: 119–127

Gastrointestinal Tumor Study Group (1979) A multi-institutional comparative trial of radiation therapy alone and in combination with 5-FU for locally unresectable pancreatic cancer. *Am Surg* **189**: 205–211

Gastrointestinal Tumor Study Group (1987) Further evidence of effective adjuvant combined radiation and chemotherapy following curative resection of pancreatic cancer. *Cancer* **59**: 2006–2010

Gastrointestinal Tumor Study Group (1988) Treatment of locally unresectable carcinoma of the pancreas: comparison of combined modality therapy (chemotherapy plus radiotherapy) to chemotherapy alone. *J Natl Cancer Inst* **80**: 751–755

Griffin JF, Smalley SR, Jewell W, Paradelo JC, Reymond RD, Hassanein RE and Evans RG (1990) Patterns of failure after curative resection of pancreatic carcinoma. *Cancer* **66**: 56–61

Gudjonsson B (1995) Critical analysis of costs, results of resection, and the need for standardized reporting. *J Am Coll Surg* **181**: 483–503

© 2000 Cancer Research Campaign

Gralla RJ (1988) Preoperative and adjuvant chemotherapy in non small cell lung cancer. *Semin Oncol* **15**: 8–12

Hart L, Chua C and Brophy L (1989) Salvage chemotherapy for metastatic breast carcinoma using cisplatin, fluorouracil and leucovorin: a phase I/II study. *Proc Am Soc Clin Oncol* **8**: 43 (abstract)

Hoffman JP, Lipsitz S, Pisansky T, Weese JL, Solin L and Benson AB (1998) Phase II trial of preoperative radiation therapy and chemotherapy for patients with localized, resectable adenocarcinoma of the pancreas: an Eastern Cooperative Oncology Group study. *J Clin Oncol* **16**: 317–323

Hörmann K (1996) Neoadjuvante Therapiekonzepte. *Onkologie* **19**: 81–93

Kamthan AG, Morris JC, Dalton J, Mandeli JP, Chessner MR, Leben D, Cooperman A and Bruckner HW (1997) Combined modality therapy for stage II and stage III pancreatic carcinoma. *J Clin Oncol* **15**: 2920–2927

Kaplan EL and Meier P (1958) Nonparametric estimation from incomplete observations. *J Am Stat Assoc* **53**: 457–481

Klaassen DJ, MacIntyre JM, Catton GE, Engstrom PF and Moertel CG (1985) Treatment of locally unresectable cancer of the stomach and pancreas: a randomized comparison of 5-fluorouracil alone with radiation plus concurrent maintenance 5-fluorouracil – an Eastern Cooperative Oncology Group study. *J Clin Oncol* **3**: 373–378

Komaki R, Wadlers S, Peters T, Byhardt RW, Order S, Gallagher MJ, Herskovic A and Pederson J (1992) High-dose local irradiation plus prophylactic hepatic irradiation and chemotherapy for inoperable adenocarcinoma of the pancreas. *Cancer* **69**: 2807–2812

Moertel CG, Gunderson LL, Mailliard JA, McKenna PJ, Martenson JA, Burch PA and Cha SS (1994) Early evaluation of combined fluorouracil and leucovorin as radiation enhancer for locally unresectable, residual, or recurrent gastrointestinal carcinoma. *J Clin Oncol* **12**: 21–27

Moertel CG, Frytak S, Hahn RG, O'Connell MJ, Reitemeier RJ, Rubin J, Schutt AJ, Weiland LH, Childs DS, Holbrook MA, Lavin PT, Livstone E, Spiro H, Knowlton A, Kalser M, Barkin J, Lessner H, Mann-Kaplan R, Ramming K, Douglas HO, Thomas P, Nave H, Bateman J, Lokich J, Brooks J, Chaffey J, Corson JM, Zamcheck N and Novak JW (1981) Therapy of locally unresectable pancreatic carcinoma: a randomized comparison of high dose (6000 rads) radiation alone, moderate dose radiation (4000 rads + 5-fluorouracil), and high dose radiation + 5-fluorouracil. *Cancer* **48**: 1705–1710

Mohiuddin M, Regine WF, Stevens J, Rosato F, Barbot D, Biermann W and Cantor R (1995) Combined intraoperative radiation and perioperative chemotherapy for unresectable cancers of the pancreas. *J Clin Oncol* **13**: 2764–2768

Nagai H, Kuroda A and Morioka Y (1986) Lymphatic and local spread of T1 and T2 pancreatic cancer. *Ann Surg* **204**: 65–71

Nakyajima Y, Miyainoto T and Tanabes M (1979) Enhancement of mamillian cell killing by 5-fluorouracil in combination with X-rays. *Cancer Res* **135**: 185–206

Nicolson M, Webb A, Cunningham D, Norman A, O'Brien M, Hill A and Hickish T (1995) Cisplatin and protracted venous infusion 5-fluorouracil (CF) – good symptom relief with low toxicity in advanced pancreatic carcinoma. *Ann Oncol* **6**: 801–804

Parker SL, Tong T, Bolden S and Wingo PA (1996) Cancer statistics. *CA Cancer J Clin* **46**: 5–27

Popescu RA and Cunningham D (1997) Chemotherapy for advanced pancreatic cancer – some light at the end of the tunnel? *Ann Oncol* **8**: 415–416

Prott FJ, Schönekaes K, Preusser P, Ostkamp K, Wagner W, Micke O, Pötter R, Sulkowski U, Rübe C, Berns T and Willich N (1997) Combined modality treatment with accelerated radiotherapy and chemotherapy in patients with locally advanced inoperable carcinoma of the pancreas: results of a feasibility study. *Br J Cancer* **75**: 597–601

Rothman H, Cantrell JE, Lokich J, Difino S, Harvey J, Ahlgren J and Fryer J (1991) Continuous infusion 5-fluorouracil plus weekly cisplatin for pancreatic carcinoma: a Mid-Atlantic Oncology Program study. *Cancer* **68**: 264–268

Scheithauer W, Depisch D, Kornek G, et al (1994) Randomized comparison of fluorouracil and leucovorin therapy versus fluorouracil, leucovorin, and cisplatin therapy in patients with advanced colorectal cancer *Cancer* **73**: 1562–1568

Schnall SF and MacDonald JS (1996) Chemotherapy of adenocarcinoma of the pancreas. *Semin Oncol* **23**: 220–228

Spitz FR, Abbruzzes JL, Lee JE, Pisters PWT, Lowy AM, Fenoglio CJ, Cleary KR, Janjan NA, Goswitz MS, Rich TA and Evans DB (1997) Preoperative and postoperative chemoradiation strategies in patients treated with pancreaticoduodenectomy for adenocarcinoma of the pancreas. *J Clin Oncol* **15**: 928–937

Tepper JE, Nardi GL and Suit HD (1976) Carcinoma of the pancreas: review of the MGH experience from 1963–1973: analysis of surgical failure and implications for radiation therapy. *Cancer* **37**: 1519–1524

Vokes EE, Weichselbaum RR, Mick R, et al (1992) Favourable long-term survival following induction chemotherapy with cisplatin, fluorouracil, and leucovorin and concomitant chemoradiotherapy for locally advanced head and neck cancer. *J Natl Cancer Inst* **84**: 877–882

Wagener D, Rougier P, Wils J, Duez N and Selleslags J for the EORTC Gastrointestinal Tract Cooperative Group (1992) Combined radiochemotherapy for locally advanced pancreatic cancer. *Proc Am Soc Clin Oncol* **11**: 166 (abstract)

Warshaw AL and Fernandez-Del Castillo CF (1992) Pancreatic carcinoma. *N Engl J Med* **326**: 455–465

Weinerman BH and McCormick RE (1994) A phase II survival comparison of patients with adenocarcinoma of the pancreas treated with 5-fluorouracil and calcium leucovorin versus a matched tumor registry control population. *Am J Clin Oncol* **17**: 467–469

Willett CG, Lewandrowski K, Warshaw AL, Efird J and Compton CC (1993) Resection margins in carcinoma of the head of the pancreas. Implications for radiation therapy. *Ann Surg* **217**: 144–148

Yeo CJ, Cameron JL, Lillemore KD, Sitzmann JV, Hruban RH, Goodman SN, Dooley WC, Coleman J and Pitt HA (1995) Pancreaticoduodenectomy for cancer of the head of the pancreas: 201 patients. *Ann Surg* **221**: 721–733

Yeung RS, Weese JL, Hoffman JP, Solin LJ, Paul AR, Engstrom PF, Litwin S, Kowalyshyn MJ and Eisenberg BL (1993) Neoadjuvant chemoradiation in pancreatic and duodenal carcinoma: a phase II study. *Cancer* **72**: 2124–2133

*© 2000 Cancer Research Campaign* 
